# Selection and Validation of Reference Genes for Gene Expression Studies by RT-PCR in *Dalbergia odorifera*

**DOI:** 10.1038/s41598-019-39088-3

**Published:** 2019-03-04

**Authors:** Hui Meng, Yun Yang, Zhi-Hui Gao, Jian-He Wei

**Affiliations:** 1 Institute of Medicinal Plant Development, Chinese Academy of Medical Sciences & Peking Union Medical College, Beijing, 100193 China; 2 Hainan Branch Institute of Medicinal Plant Development (Hainan Provincial Key Laboratory of Resources Conservation and Development of Southern Medicine), Chinese Academy of Medical Sciences & Peking Union Medical College, Haikou, 570311 China

## Abstract

Perennial tree *Dalbergia odorifera* T. Chen could form the precious heartwood used to produce chinese traditional medicine, rosewood furniture and fragrances. However the formation of heartwood is time-consuming and low efficient, leading to the severe destruction of its wild resources. Thus, it is urgent to study the molecular mechanism of heartwood formation in *D*. *odorifera*. But till now, there is no report about the reference gene selection in this species. In this study, the expression stability of nine candidate reference genes were evaluated across different tissues and stems treated by wound and chemical stimulators. Four algorithms were applied to obtain the robust genes. The results support *HIS2*, *GAPDH*, and *CYP* to be the most stable reference genes in samples under different wound treatments while *DNAj* was the least stable. In different tissues, *HIS2*, *UBQ*, and *RPL* were the most stable reference genes while *DNAj* was the least stable. The selected reference genes were validated through the normalization of the qRT-PCR data of six heartwood related genes in terpene biosynthesis pathway and ethylene signal pathway. The results showed that their expression levels were accurate when they were normalized by the most stable reference gene *HIS2*, or by the combination of the two or three most stable reference genes. These results demonstrated that these selected reference genes are reliable.

## Introduction

Rosewood is the heartwood from species of genera *Pterpcarpus*, *Dalbergia*, *Diospyros*, *Millettia* and *Cassia* which mainly grow in tropical area^1^. Because of its roughness and pleasing appearance, rosewood is always used in high-valued furniture and artware production. Among rosewoods from all 29 species, the rosewood of *Dalbergia odorifera* T. Chen which is indigenous in Hainan, PR China is the most precious^2,3^. Besides to be used as rosewood to produce furniture, the heartwood of *D*. *odorifera* could also be used as “Jiangxiang” to produce chinese traditional medicine because of its obvious medicinal effects on symptoms such as blood disorder, ischemia, swelling, necrosis and rheumatic pain^4^. Just in traditional chinese medicine market, the annual demand of Jiangxiang raw materials has reached to about 250–300 tons. Due to the high price and large demands, nowadays, the wild resource of *D*. *odorifera* has been greatly reduced. In Hainan Province, the number of *D*. *odorifera* trees over 30 years old is less than 50. *D*. *odorifera* has been listed on the IUCN red list as endangered species^5^ and the Appendix II list of the Convention on International Trade in Endangered Species of Wild Fauna and Flora since 2007^6^.

Previous phytochemical studies demonstrated that the main compounds of the heartwood included phenolic compounds, flavonoids and volatile oil (mainly terpenoids, which account for more than 60%), which showed a variety of biological activities, such as antitumor, antiinflammatory, antianalgesic, antioxidant, antiplasmodial, antinephritic, neuroprotective, antimicrobial, and antiplatelet activities^7–12^. Nowadays, the volatile oil is the major material used in chinese traditional medicine.

The formation of heartwood is regulated temporally and spatially, which is a long and complex physiological process^13,14^. The *D*. *odorifera* trees grow very slowly, and their heartwood formation is much slower. Generally, the trunk begins to form heartwood after growing for 8–10 years. And it takes at least 30 years before it can be used as timber. In the natural environment, heartwood is formed mainly in the center of the trunk and root. In addition, when affected by certain external factors, such as mechanical damage, insect attack, microbial invasion, phytohormones, or drought, the trees would also produce heartwood around the wound or rotting parts of the trunk or root. Recent studies have shown that *D*. *odorifera* can be induced to produce heartwood by ethylene, 6-benzylaminopurine (6-BA), drought, microorganism and mechanical damage^15–18^. Thus, to improve heartwood production, it is urgent to study the exact mechanisms underlying its formation.

Gene expression analysis is a universal and fundamental method to explore the molecular mechanisms of various biological processes. Even though several large-scale gene expression tools have been developed, the quantitative real-time PCR (qRT-PCR) assay with its advantages on the high sensitivity, accuracy, specificity, and good reproducibility is normally used as the small-scale tool in gene expression analysis^19,20^. To avoid the variations caused in each step of the entire experimental procedure^21^, the internal reference genes are crucial in normalization of the qRT-PCR data. The ideal reference gene should express at stable level in different cell types and under different experimental conditions^22^. However, the expression levels of some widely used reference genes have also been proved to be varied across different samples or under different treatments^23–26^ and no universal reference genes have been identified until now. Meanwhile, more and more studies have focused on the evaluation of the expression stability of potential reference genes in special species or under specific conditions. Several algorithms have been developed to validate the performances of candidate reference genes. The geNorm software conducts pair-wise comparison and calculates the M value of all candidate genes^27^. Low M value represents a slight variant. Furthermore, the pair-wise variation (V_n_/V_n+1_) between the sequential normalization factors (NF) (NF_n_ and NF_n+1_) were calculated to determine the optimal number of reference genes required for accurate normalization. NormFinder is a model based program designed to calculate stability value according to the intra-group and inter-group variation^28^. The candidates are ranked according to their expression stability. The lower the stability value, the more stable the reference genes. BestKeeper determines the “optimal” reference genes using pair-wise correlation analysis of all pairs of candidate genes^29^. The deltaCt method compares relative expression of pairs of reference genes within each sample^30^.

However, no report is available concerning reference gene selection in *D*. *odorifera*, especially for its heartwood formation by different wound treatments. In this study, the expression stability of nine candidate reference genes were assessed in *D*. *odorifera* samples exposed to different wound treatments or in different tissues using geNorm, Normfinder, Bestkeeper, and deltaCt algorithms. The combined results were calculated using refFinder. The relative expression of six genes including genes in the terpene biosynthesis pathway and ethylene signal pathway were used to validate the reliability of the selected reference genes and to compare the effect of different wound treatments.

## Results

### Candidate reference gene selection and primer design

Candidate reference genes were selected according to the previous reports in other plants or the expression pattern in *D*. *odorifera* transcriptome data^31–34^. Ortholog sequences of these genes in *D*. *odorifera* were obtained in the 454 high-throughput sequencing library and identified through BLASTX against GenBank. According to the identity of the annotation of each gene with its ortholog, we developed a list of nine candidate genes including those encoding actin (*ACT*), polyubiquitin (*UBQ*), ribosomal protein (*RPL*), glyceraldehydes-3-phoshpate (*GAPDH*), chaperone protein dnaJ (*DNAj)*, histone H2B (*HIS2*), elongation factor 1-alpha (*EF1*), peptidyl-prolyl cis-trans isomerase (*CYP*) and 18S ribosomal RNA (*18S*) (Table 1). Agarose gel electrophoresis was performed and the amplification specificity of each primer pair was confirmed by a single band with the desired size of each amplicon. The melting curve analysis of qRT-PCR results also proved the primer specificity with a single peak for each product. PCR products were further confirmed by sequencing and BLAST against GenBank and the EST library.

**Table 1:** Details of primers and amplicons of nine candidate reference genes.

Gene symbol	Gene name	Primer Sequence (5′-3′)	Amplicon length	Annealing Temp	PCR efficiency
*GAPDH*	glyceraldehyde-3-phosphate dehydrogenase	F: AGGAGTCTGAAGGCAAGTTGAA	181 bp	55	93%
		R: CGCGAGTGCTGTAACCC			
*ACT*	Actin	F: CAATGAATTGCGTGTTGCT	182 bp	55.4	98%
		R: ATACCAGTTGTGCGACCACTT			
*UBQ*	Ubiquitin	F: GGTCGTACTTTGGCAGACTACA	248 bp	52.4	96%
		R: CCCAGAATCATCACACGGAC			
*DNAJ*	chaperone protein DNAj	F: GCCTCCCTATCAACGACAGA	163 bp	60	108%
		R: AGCAGTGGTATCAACGCAGA			
*HIS2*	Histone H2B	F: TGGCCAAGCACGCTGTT	210 bp	52.7	105%
		R: TGCACAAAAAGGATCGAGCTGA			
*EF1*	elongation factor 1-alpha	F: TCACATTAAAGCCAACATTATCA	222 bp	54.2	99%
		R: AGAGACCCACAGACAAGCCT			
*18* *S*	18S rRNA gene	F: CCCGTCGCTCCTACC	138 bp	55.2	97%
		R: CGGAAACCTTGTTACGACTT			
*CYP*	Peptidyl-prolyl cis-trans isomerase	F: AACGCAGAGTCCATCTACGG	191 bp	58.4	103%
		R: TTCATGCCCTCCACGATCT			
*RPL*	60S ribosomal protein L10	F: TATGCCTGATGGTGTGAATGC	228 bp	53.8	94%
		R: TGGAAGGTGGCTTGAGTCG			

### Expression variation of candidate reference genes

**Figure 1:**
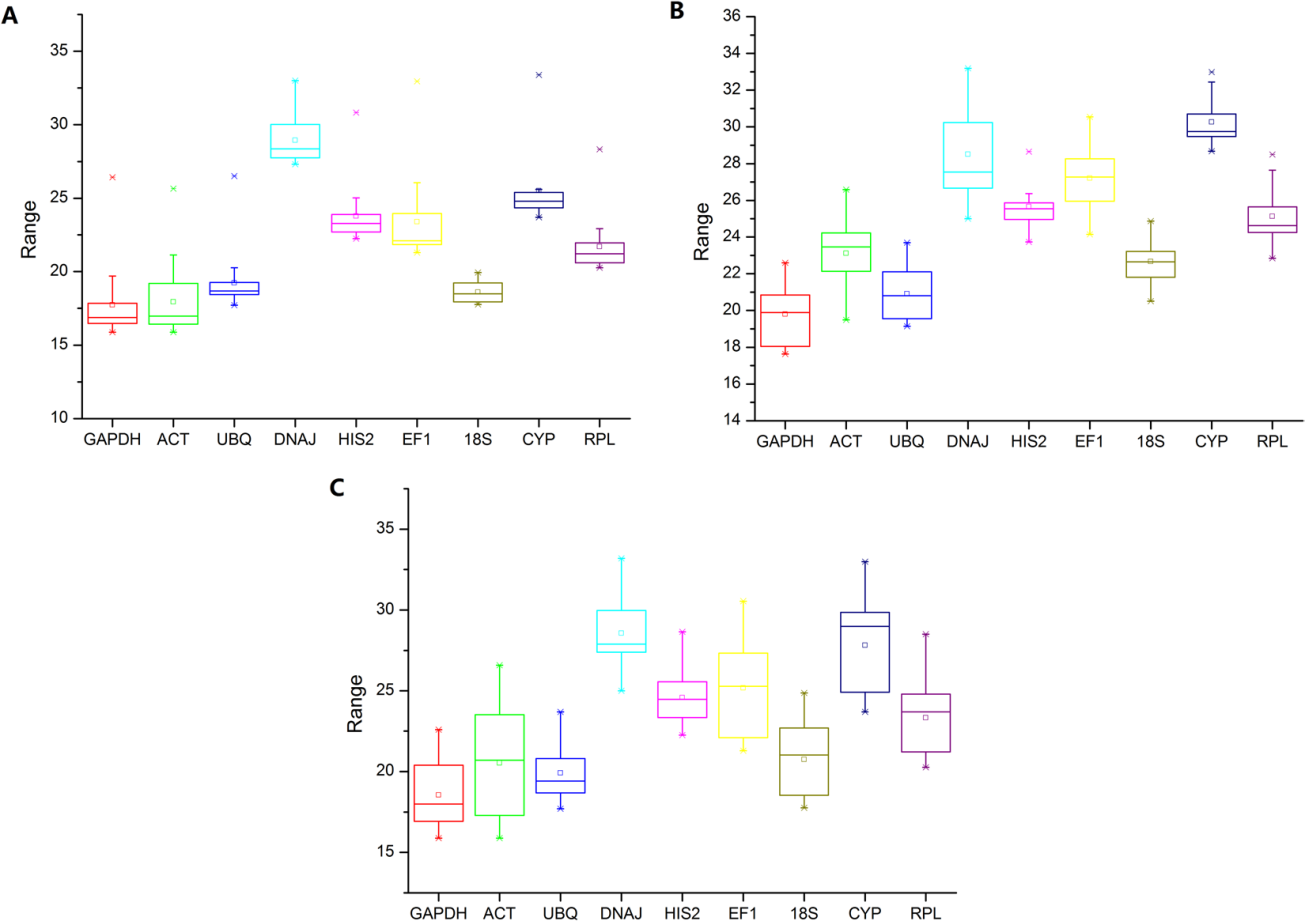
Variation of qRT-PCR Ct values for all candidate reference genes. Box charts of Ct value for each reference gene in samples under different wound treatments (**A**), different tissues (**B**), or in all samples (**C**). The horizontal line and little square in the box show the median values and mean value respectively, surrounded by lower and upper boxes indicating the first and third quartile. The vertical lines indicate the value ranges.

To obtain the most reliable results, the quality of isolated total RNAs was assessed by strict quality control method. OD 260/280 value examined by Nano drop ND2000 ranged between 2.0 and 2.2. Electrophoresis showed sharp and intense 18S and 28S ribosomal RNA bands with a practical absence of smears. The primary cDNA template amount used in all real-time PCR reactions was determined by the standard curves of all candidate reference genes to make sure the threshold cycle (Ct) for each one kept in an appropriated range (15–35). Finally, the average Ct value of all genes ranged from 15 to 34 (Figure. 1). Amongst all the genes, *GAPDH*, *UBQ*, *ACT*, and *18S* were the most abundant with the average Ct value close to 20. For all the tested genes, their expression levels in two subgroups were similar, however, their expression variation in different tissues were higher than that in different wound treatments, and the overall variation was much higher than that of each subgroup. In different tissue subgroup, *HIS2* and *CYP* showed a comparatively narrower range of expression variation, while in different wound treatment subgroup, more genes showed little expression variation, indicating a possibility that different reliable reference genes might be selected for two subgroups.

### Expression stability of the candidate reference genes in different tissues

In order to minimize the bias generated by the assumptions underlying each evaluation method, the expression data were analyzed by four different statistical approaches to rank the stability of candidate housekeeping genes.

The results of geNorm analysis showed that, for different tissues, all candidate genes reached high expression stability with relatively low M values far below the default limit of 1.5 (Table 2). Among them, *HIS2* and *RPL* were the most stable genes with least M values of 0.541 and followed by *CYP* and *UBQ*, with the M value of 0.589 and 0.841 respectively.

**Table 2:** Ranking of the candidate reference genes in different tissues using geNorm, Normfinder, Bestkeeper, and delta Ct algorithms.

Rank	geNorm		NormFinder		BestKeeper		deltaCt		Comprehensive Ranking	
	Gene	SV	Gene	SV	Gene	SV	Gene	SV	Gene	Geomean Ranking Value
1	*HIS2*	0.541	*UBQ*	0.388	*HIS2*	0.87	*UBQ*	0.97	*HIS2*	1.57
2	*RPL*	0.541	*GAPDH*	0.614	*18S*	0.88	*HIS2*	1.05	*UBQ*	2.11
3	*CYP*	0.589	*HIS2*	0.651	*CYP*	0.92	*GAPDH*	1.05	*RPL*	3.46
4	*UBQ*	0.814	*EF1*	0.663	*RPL*	1.05	*EF1*	1.1	*GAPDH*	3.81
5	*GAPDH*	0.887	*CYP*	0.747	*UBQ*	1.2	*CYP*	1.11	*CYP*	3.87
6	*EF1*	0.929	*RPL*	0.809	*GAPDH*	1.39	*RPL*	1.13	*EF1*	5.26
7	*18S*	1	*ACT*	1.127	*ACT*	1.39	*ACT*	1.36	*18S*	5.47
8	*ACT*	1.082	*18S*	1.152	*EF1*	1.5	*18S*	1.37	*ACT*	6.96
9	*DNAJ*	1.189	*DNAJ*	1.370	*DNAJ*	2.17	*DNAJ*	1.56	*DNAJ*	9

The NormFinder algorithm demonstrated a different reference gene of choice. According to the stability value calculated by this software, *UBQ* with the least stability value of 0.388 ranked top as the best reference gene, followed by *GAPDH* (Table 2).

Because the BestKeeper program is designed to assess the reliability of the reference genes and determine a reliable normalization factor, but not to rank their reliability, we listed the candidate genes according to their standard deviation (SD) value. The BestKeeper program calculated *HIS2*, *18S*, and *CYP* as the most suitable genes in all tissue samples. The results of deltCt method showed that in different tissues, *UBQ*, *HIS2*, and *GAPDH* were the most stable genes with least SD value, while *DNAj* and *18S* were the least stable genes.

With the purpose to identify the robust reference genes of all tested samples in *D*. *odorifera*, we applied RefFinder algorithm (http://150.216.56.64/referencegene.php) to aggregate all lists generated by each of the four approaches. RefFinder bases on the ranking from each program. It assigns an appropriate weight to an individual gene and calculated the geometric mean of their weights for the overall final ranking. The aggregation list obtained showed that *HIS2*, *UBQ* and *RPL* were the top three reliable reference genes. In contrast, *DNAj*, *ACT*, and *18S* were the lowest ranked and should be avoided as the normalization gene (Table 2).

### Expression stability of the candidate reference genes after different treatments

The geNorm program calculation showed that for different treatments, all genes are qualified too, and the most stable genes are *GAPDH* and *CYP* with the least M values of 0.354, and followed by *HIS2* and *RPL*, with M values of 0.485 and 0.559 respectively (Table 3).

The result of NormFinder analysis showed that *HIS2* and *GAPDH* had the least variation value of 0.318 and 0.381 respectively in the subset of different wound treatments.

The BestKeeper program calculated *UBQ*, *18S* and *HIS2* as the most suitable genes in samples with different treatments, whereas *DNAj* and *18S* were the least reliable (Table 3). The results of deltCt method showed that *HIS2*, *GAPDH* and *CYP* were the most stable genes, while *DNAj* and *ACT* were the least stable genes.

**Table 3:** Ranking of the candidate reference genes in samples under different treatments using geNorm, Normfinder, Bestkeeper, and delta Ct algorithms.

Rank	geNorm		NormFinder		BestKeeper		deltaCt		Comprehensive Ranking	
	Gene	SV	Gene	SV	Gene	SV	Gene	SV	Gene	Geomean Ranking Value
1	*GAPDH*	0.354	*HIS2*	0.318	*UBQ*	0.41	*HIS2*	0.83	*HIS2*	1.73
2	*CYP*	0.354	*GAPDH*	0.381	*18S*	0.6	*GAPDH*	0.84	*GAPDH*	2.11
3	*HIS2*	0.485	*CYP*	0.488	*HIS2*	0.62	*CYP*	0.89	*CYP*	2.71
4	*RPL*	0.559	*UBQ*	0.529	*RPL*	0.64	*RPL*	0.91	*UBQ*	3.16
5	*UBQ*	0.611	*RPL*	0.549	*GAPDH*	0.74	*UBQ*	0.92	*RPL*	4.23
6	*18S*	0.759	*EF1*	0.892	*CYP*	0.78	*EF1*	1.13	*18S*	4.92
7	*EF1*	0.856	*18S*	0.901	*DNAJ*	1	*18S*	1.16	*EF1*	6.7
8	*ACT*	0.927	*ACT*	1.045	*EF1*	1.17	*ACT*	1.23	*ACT*	8.24
9	*DNAJ*	1.039	*DNAJ*	1.287	*ACT*	1.17	*DNAJ*	1.43	*DNAJ*	8.45

The final list obtained by refFinder showed that *HIS2*, *GAPDH* and *CYP* were the top three reliable reference genes. In contrast, *DNAj*, *ACT*, and *EF1* were the lowest ranked and should be avoided as the normalization gene (Table 3).

**Figure 2:**
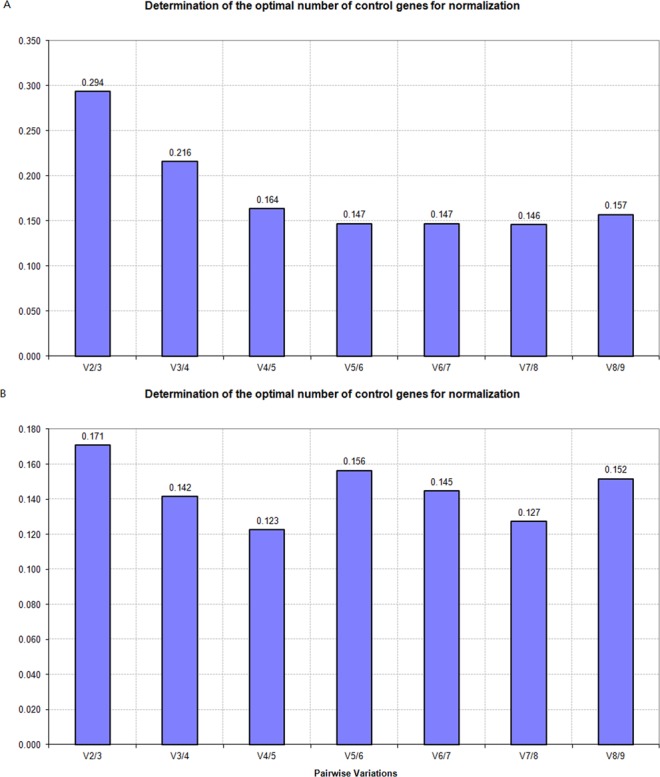
GeNorm calculated the minimum number of reference genes necessary for reliable and accurate normalization. Determination of the optimal number of reference genes for qRT-PCR normalizationin different tissues (**A**), stem tissues treated by different wounds (**B**). Pair-wise variation values (V_n/n+1_) were measured between two sequential normalization factors NFn and NF_n+1_ by geNorm. A, V_n/n+1_ values for different tissue samples; B, V_n/n+1_ values for samples with different wound treatments.

geNorm was also adopted to determine the optimal number of reference genes required for accurate normalization. For all tissue samples, the V_5/6_ was 0.147 (Figure. 2), indicating that including the five most stable reference genes might be best for the accurate analysis. For different wound treatments, the V_3/4_ was 0.142 which is lower than the threshold of 0.15, demonstrating that including the three most stable reference genes, *HIS2*, *GAPDH* and *CYP*, might be sufficient for the accurate analysis. However, taking all samples together, none of the V_n/n+1_ value could reach the standard <0.15, demonstrating that it’s hard to identify reference genes for both sample subgroups.

### Validity of selected reference genes

To assess the validity of the selected reference genes, expression of potential heartwood related genes in terpene biosynthesis pathways and ethylene response pathways were normalized using the most stable gene or least stable gene. These genes have been identified as wound up-regulated genes by the transcriptome data (SP Table 1).

**Figure 3:**
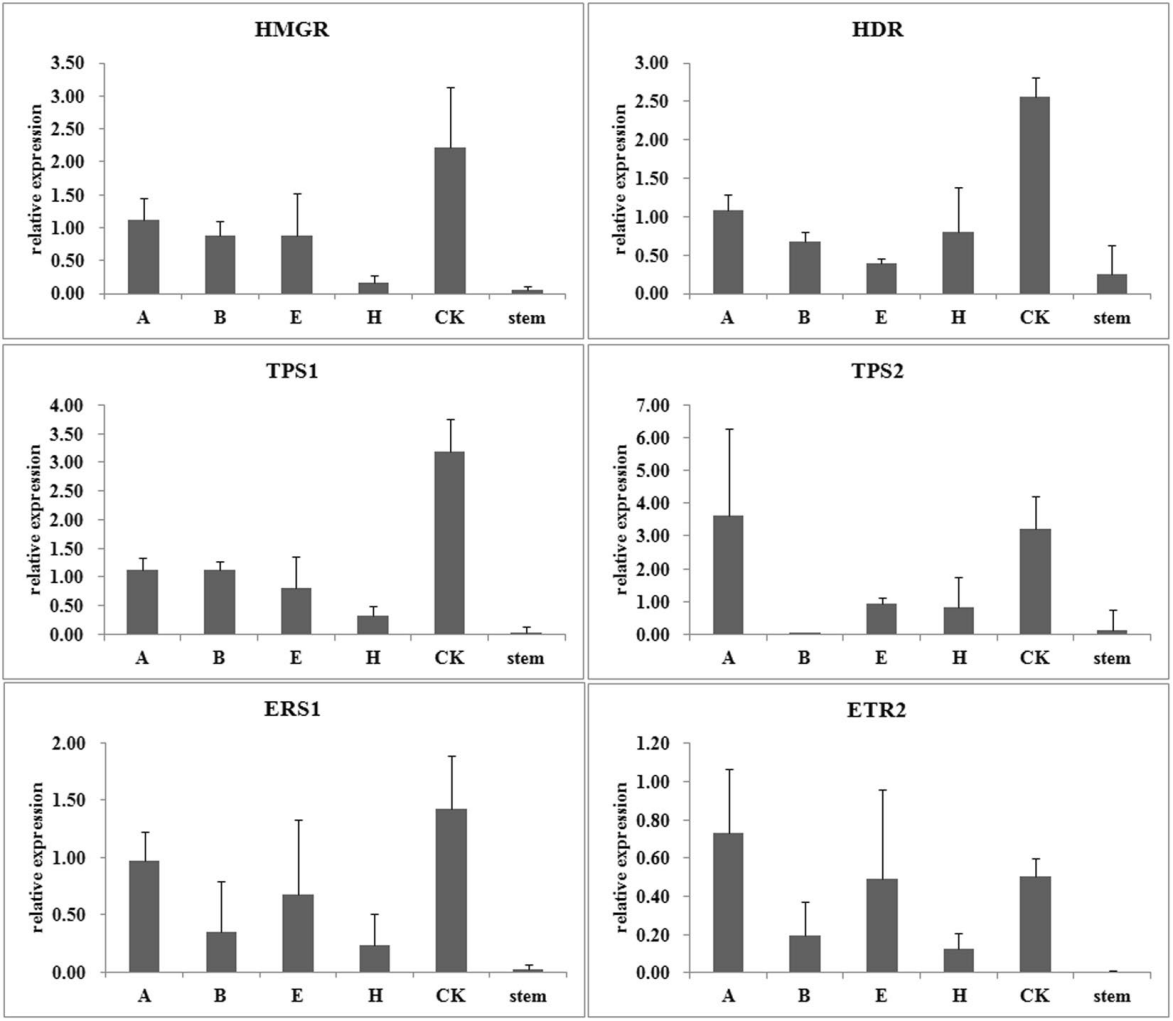
Relative expression levels of heartwood related genes normalized by the unstable reference gene *18S*. The expression levels in stems treated with wound and different phytohormones were normalized to by *18S*. A, ABA; B, 6-BA; E, ethylene; H, H_2_O_2_; CK, ddH_2_O.

**Figure 4:**
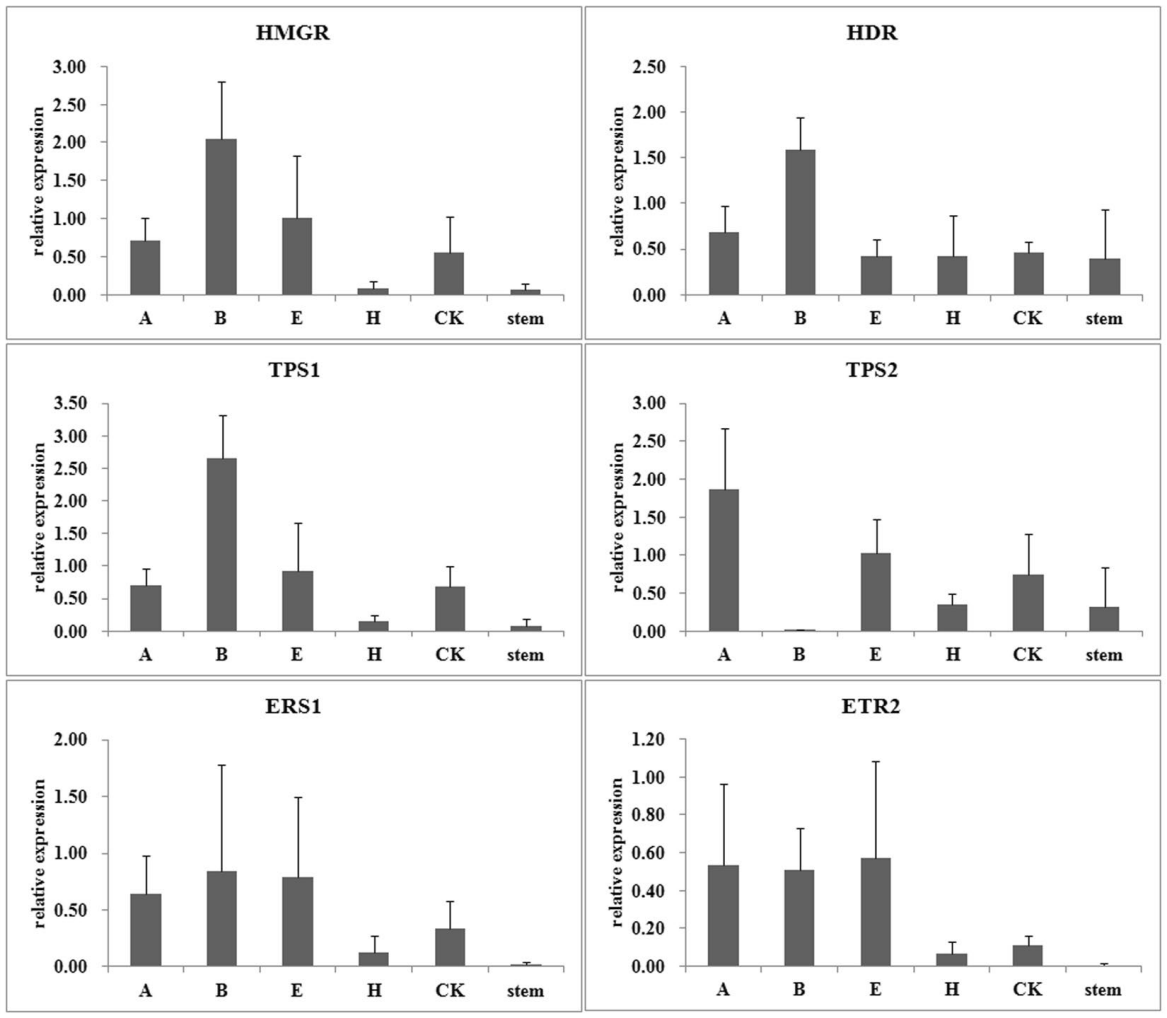
Relative expression levels of heartwood related genes normalized by the least stable reference gene *DNAj*. The expression levels in stems treated with wound and different phytohormones were normalized to by *DNAj*. A, ABA; B, 6-BA; E, ethylene; H, H_2_O_2_; CK, ddH_2_O.

**Figure 5:**
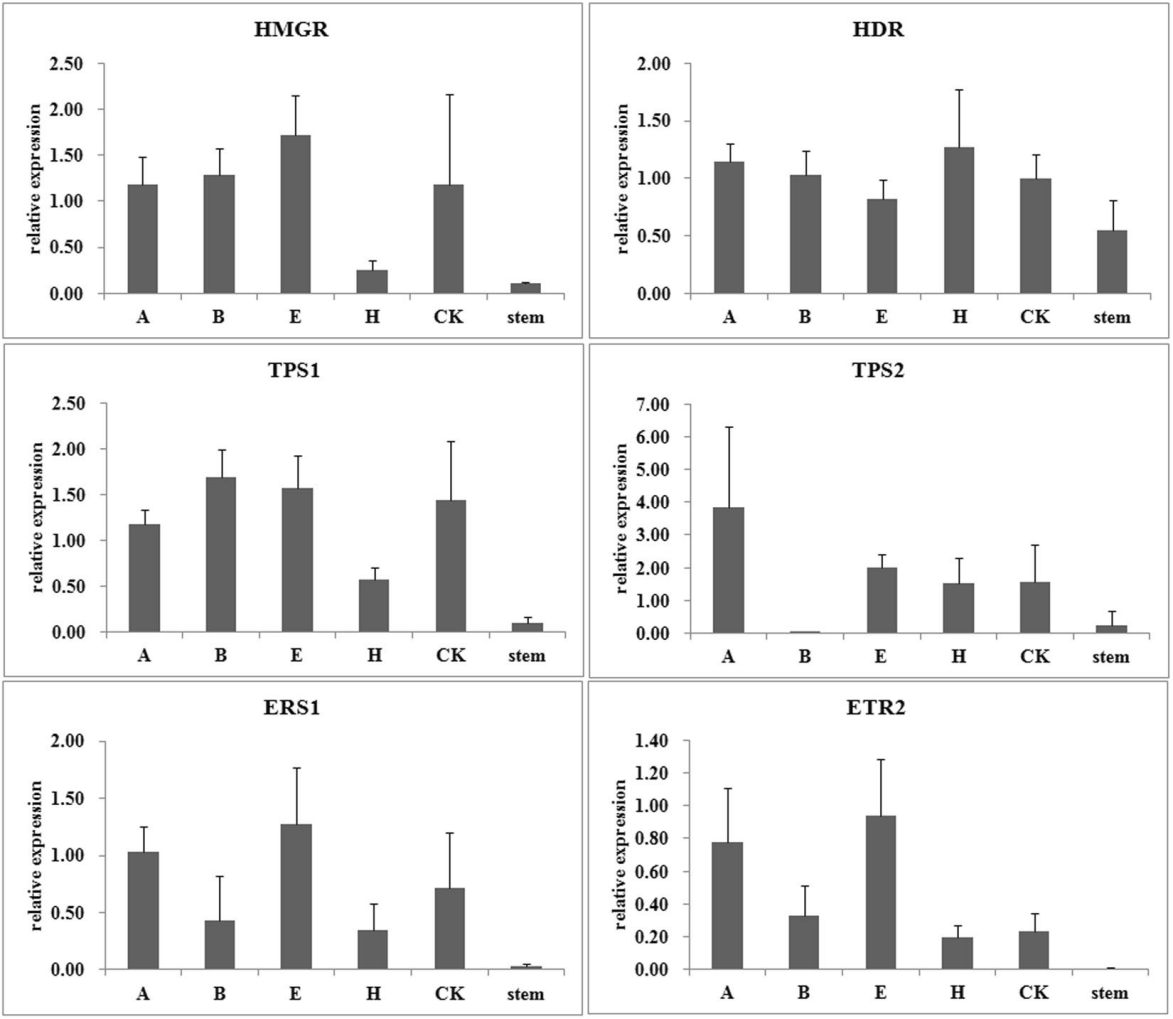
Relative expression levels of heartwood related genes normalized by the most stable reference gene *HIS2*. The expression levels in stems treated with wound and different phytohormones were normalized to by *HIS2*. A, ABA; B, 6-BA; E, ethylene; H, H_2_O_2_; CK, ddH_2_O.

The up-regulation of all these tested genes by different wound treatments could be confirmed, irrespective of the reference genes used (Figures 3–5). However, the detailed expression level was obviously different. For example, when *18S* was used to normalize the expression data, almost all genes exhibited the highest expression level in the CK sample with ddH_2_O treated on the wound (Figure. 3). This is inconsistent with our heartwood production experiment. Another unusual phenomenon was observed when the least stable gene *DNAj* was used as reference gene. The expression levels of many genes in the samples with H_2_O_2_ treated on the wound were less than those in the CK samples (Figure. 4). Furthermore, when the top ranked two or three reference genes were adopted, the expression patterns of these tested genes are highly similar (Figure. 5, SP Figs 1 and 2). These results demonstrated that the reference gene selected could influence the accuracy of gene expression level in the qRT-PCR data analysis and confirmed that the top ranked *HIS2* was more reliable than the least ranked genes as the reference gene. The result reinforces the significance of validating reference genes prior to experimental applications.

## Discussion

The precious heartwood of *D*. *odorifera* is formed extremely slowly and mainly in the centre of trunks and roots naturally. However, the demand in the market is growing. For the purpose of obtaining the heartwood, natural resource of *D*. *odorifera* is being destroyed. To solve this problem, researchers have paid great attention to the efficient production of heartwood. In recent years, researchers have noticed that different wound and biotic stresses could induce the heartwood formation in tissues outside of the wood centre. To reveal the secret of heartwood formation, it is urgent to conduct researches on the molecular mechanisms of the induced heartwood formation and identify important genes in this process. Thus, identification of suitable reference genes in *D*. *odorifera*, especially in samples under different treatments, is necessary and critical for accurate gene expression assessment.

Several algorithms have been developed for reference gene selection. Because each algorithm has its own bias, we applied the four algorithms to evaluate the candidate reference genes, and selected the most reliable ones in *D*. *odorifera*, especially for studying the mechanism of heartwood formation or the biosynthesis of major components. The analysis by the four programs generated slightly different ranking. Since these approaches are intrinsically biased, this result is not beyond our expectation. In order to counteract this bias influence, we applied the refFinder to aggregate the four gene lists. Our result demonstrated that for different wound treatments, *HIS2*, *GAPDH*, and *CYP* are the most reliable reference genes and for different tissues, *HIS2*, *UBQ*, and *RPL* are the three most reliable reference genes. Histone encoding genes are commonly used reference genes in many species and tissues. Even though it has been reported to be unstable in several studies^35,36^, it has also been proved to be the choice in some other reports. For instance, study in a dominant species of desert ecosystems *Reaumuria soongorica* showed that histone *H2A* was one of the most stable reference genes under 4 kinds of abiotic stresses (drought, salt, dark, and heat)^37^. Another histone gene *HIS3* and *eIF4A* were identified as the most suitable for analyses of abiotic stress in tissues of annual ryegrass^38^. In green alga cultured in various environments, histone *H4* was one of the most suitable^39^. These results implied an advantage of histone genes as reference gene in plants response to environmental factors and stresses. Besides, our result here also demonstrated that histone genes might be used in different tissues. The other stable gene in different treatment subgroup, *GAPDH*, is one of the most commonly used reference genes in many species and tissues, although it has been reported to be unstable in several studies^26,40,41^. Our previous study in *Aquilaria sinensis* has showed that it was one of the most stable genes under different treatments^23^. It was one of the top-ranked reference genes in different tissues or for cold or sucrose stresses in Chinese tallow^42^ and was identified as the most stably expressed gene in flax^43^. In addition, it was selected as one of the stable reference genes for “hormone stimuli” group in carrot leaves^44^, and was a suitable reference gene under salinity/drought-treatment in sugarcane^45^. These results indicated the potential of *GAPDH* for being employed as the reference gene for a wide range of purposes.

Another stable reference gene *UBQ* was found to be one of the most stable genes for the different cultivars and different tissues examined, and for fruit developmental stages in the food seasoning Chinese prickly ash^46^. In a wide variety of stages of longan cultured under different temperatures, it was identified as one of the internal controls for gene expression analysis^35^. And it was one of the top-ranked reference genes as well as *GAPDH* in all samples in Chinese tallow^42^.

We demonstrated the fidelity of the selected reference genes *HIS2*, by normalizing the relative expression of several heartwood related genes in *D*. *odorifera*. These genes were identified from our 454 transcriptome sequencing data by BLASTX, and all of them were proved to be up-regulated by wound treatment according to the transcriptome study. 3-hydroxy-3-methylglutaryl-CoA reductase gene (*HMGR*), (E)-4-hydroxy-3-methylbut-2-enyl diphosphate reductase (*HDR*), terpene synthase genes (*TPS*) were genes encoding enzymes of plant terpene biosynthesis pathway^47,48^. In the validation of *HIS2* as reference gene, it was confirmed that they could be induced by wound, especially for *HMGR*, *TPS1*, and *TPS2*. The induction of *HDR* was not as obvious as them, this is because that *HDR* expressed at a moderate level in untreated stems, whereas the other three almost had no expression at all. Since *HMGR* and *HDR* are genes from MVA and MEP pathways respectively. This demonstrated that the terpenes in heartwood might be synthesized by the MVA pathway. This result is consistent with some other researches which also showed that wound could induce the terpene biosynthesis through MVA pathway^49^. Moreover, we noticed that the expression of two *TPS* genes had different expression pattern. After 6-BA treatment, *TPS1* could be obviously induced, whereas *TPS2* was totally inhibited. Although the deep reason under this need to be further studied, this demonstrated that different treatments might influence the components of the heartwood because different TPSs always produce different terpenes.

ERS1 and ETR2 are two ethylene receptors in Arabidopsis, and play very important role in ethylene signal transduction. Our results were consistent with researches in Arabidopsis showed that they could be induced by ethylene^50,51^. The wound and ethylene induction of their expression proved by this study demonstrated that these two genes might function as ethylene receptors in *D*. *odorifera* too and take part in the heartwood formation. Furthermore, when *18S* was used as reference gene, the plant hormone treatments were all shown to have negative effect on the expression of these genes, compared with CK which only treated by wound and ddH_2_O. This is not consistent with our and other heartwood induction experiments. As many other studies, our study proved that if the unsuitable reference gene was used, the data analysis would result in biased result.

## Methods

### Plant materials and treatments

All samples were collected from seven-year-old *D*. *odorifera* T. Chen trees in the planting base of Hainan Branch, the Institute of Medicinal Plant Development of China. For different tissues, roots, stems, leaves, flowers, and fruits were cut off by scalpels or gardening scissors. For different wound treatments, stems were incised by single side razor blades to produce 4 × 2 × 0.5 cm wound. The wounds were then treated with 1% H_2_O_2_, ethephon, 6-BA, or abscisic acid (ABA) for 24 hours. The mock-treated stems with only ddH_2_O supplemented on the wound for the same time interval served as the control. All samples were collected in biological triplicate. All samples were ground in liquid nitrogen for RNA isolation or stored at −80 °C until use.

### Total RNA isolation and cDNA synthesis

Total RNA was extracted from all samples using the RN53 EASYspin Plus Total RNA Extraction Kit (AIDLAB) according to the manufacturer’s instruction and then was treated with RNase-free DNase I (TaKaRa, Japan). The RNA integrity was checked by 1% agarose gel electrophoresis. The quantity and quality of each RNA sample were examined by a NanoDrop ND-2000 Spectrophotometer (Thermo Fisher, USA) to confirm the OD260/OD280 value between 2.0 and 2.2. 1 μg RNA for each sample was used in the 25 μl reverse transcription reaction system with RevertAid™ first strand cDNA Synthesis kit K1622 (Thermo Scientific) according to the manufacturer’s instruction.

### PCR primer design

A total of nine commonly used housekeeping genes were selected and ortholog sequences from the EST library of *D*. *odorifera* were used for primer design. Detailed information of these genes is described in Table 1. Primers were designed with the EST sequences by Oligo 6 with PCR amplicon length of 100–300 base pairs (Table 1).

### Quantitative real-time PCR and amplification efficiency test

The qRT-PCR reaction was performed using ABI SYBR Selected Master Mix kit (4472908) and run on 96-wells plates with the Applied Biosystem StepOnePlusTM Real-Time System. 2 μl template cDNA was added to the 12.5 μl PCR reaction mixture containing 0.2 μM of each primer. ddH_2_O was used to compensate the reaction system to a final volume of 25 μl. PCR was initiated with 1 minute incubation at 95 °C and followed by 40 cycles of 95 °C for 15 seconds and 60 °C for one minute. All qRT-PCR reactions were carried out in biological and technical triplicates. Each PCR was repeated three times. The specificity of the qRT-PCR reactions was determined by melt curve analysis of the amplified products with a heating procedure from 72 °C to 95 °C in 0.5 °C steps. Negative controls with water instead of cDNA were included for each gene target under assay.

Primer efficiencies and standard deviations were calculated based on a standard curve generated using a tenfold dilution of template cDNA over at least four dilution points that were measured in triplicate. A variation of 10% was allowed. Amplicon specificity was confirmed by sequencing.

The amplification products were sequenced and BLAST against GenBank and our EST library of *D*. *odorifera* to confirm whether they were the target gene sequences.

### Data analysis

For geNorm and NormFinder analysis, expression level was calculated relative to the sample with the highest expression. For BestKeeper analysis, the Ct values were input directly to the Excel table. Analysis methods followed the manufacturer’s instruction. The combined rank was generated using the refFinder algorithm.

### Relative expression of heartwood related genes

All qRT-PCR reactions were carried out in biological and technical triplicates. The comparative 2^ΔΔCt^ method was used to evaluate the relative quantities of each amplified products.

## Supplementary information

SP figure

Supplementary Dataset 1
