# Supplementary material for: Selection and Validation of Reference Genes for Gene Expression Studies by RT-PCR in Dalbergia odorifera
Source: Sci Rep. 2019 Mar 4;9:3341. doi: 10.1038/s41598-019-39088-3 (PMC6399326; doi:10.1038/s41598-019-39088-3)
Supplement: Supplementary file 1 — SP figure [file 41598_2019_39088_MOESM1_ESM.docx]

Selection and Validation of Reference Genes for Gene Expression Studies by RT-PCR in *Dalbergia odorifera*

Hui Meng^1,2^, Yun Yang^1,2^_,_ Zhi-Hui Gao^1*^, Jian-He Wei^1,2*^

1Institute of Medicinal Plant Development, Chinese Academy of Medical Sciences & Peking Union Medical College, Beijing 100193, China

2Hainan Branch Institute of Medicinal Plant Development (Hainan Provincial Key Laboratory of Resources Conservation and Development of Southern Medicine), Chinese Academy of Medical Sciences & Peking Union Medical College, Haikou, 570311, China

^*^Corresponding author

Tel: +86 10 57833358

Fax: +86 10 57833358

Email addresses

J.W.: [wjianh@263.net](mailto:wjianh@263.net)

Z.G.: [huihuigao@163.com](mailto:huihuigao@163.com)


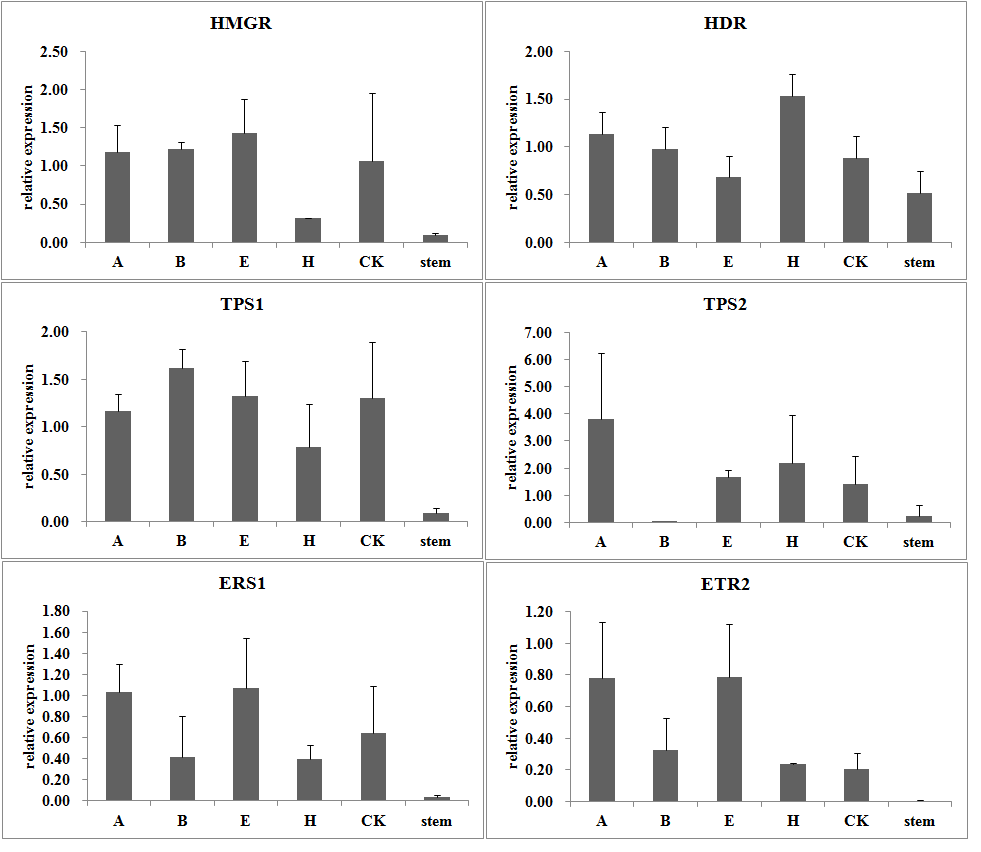


## SP Figure 1. Relative expression levels of heartwood related genes normalized by the two most stable reference gene *HIS2* and *GAPDH*.

The expression levels in stems treated with wound and different phytohormones were normalized to by *HIS2* and *GAPDH*. A, ABA; B, 6-BA; E, ethylene; H, H_2_O_2_; CK, ddH_2_O.


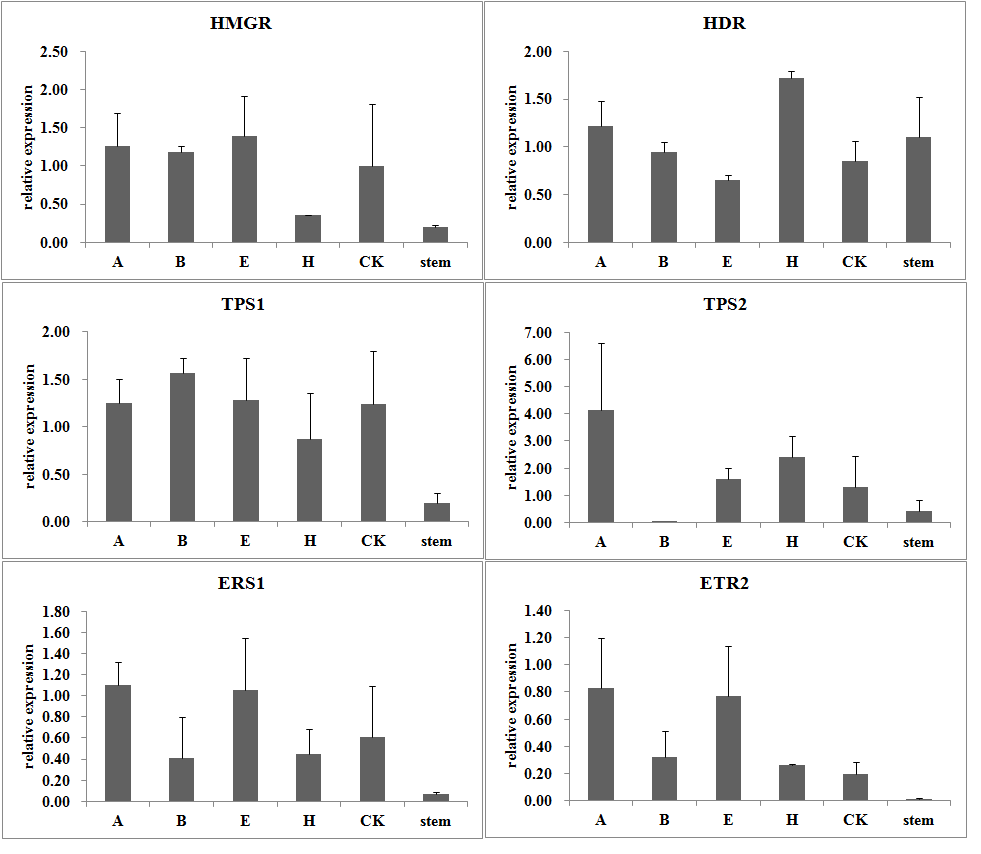


## SP Figure 2. Relative expression levels of heartwood related genes normalized by the three most stable reference gene *HIS2*, *GAPDH*, and *CYP*.

Theexpression levels in stems treated with wound and different phytohormones were normalized to by *HIS2, GAPDH*, and *CYP*. A, ABA; B, 6-BA; E, ethylene; H, H_2_O_2_; CK, ddH_2_O.

**SP Table 1. Information of the six genes used in reference gene validation**
